# Pilocarpin in Toothache

**Published:** 1886-03

**Authors:** 


### Pilocarpin in Toothache.

The *Medical Press and Circular* says that Dr. Kurzakoff, of Moscow, claims that hypodermic injections of pilocarpin relieved toothache very quickly in several cases in which he tried it:

A solution of two grains of the salt in half an ounce of distilled water was used, the injection being made into the temporal region on the side of the odontalgia. In two of the cases one-eighth, and in a third case one-quarter of a grain of the salt was injected. In all the cases pain disappeared permanetly in about an hour after the injection ; about the same time salivation and perspiration (caused by the drug) also ceased. In one of the cases, in that of a man, aged forty-six, with rheumatic periodontitis associated with agonizing earache, the injections (of a quarter of a grain) produced profuse vomiting, with cyanosis, general weakness, and drowsiness, all of which symptoms disappeared in about an hour an half after taking twenty drops of tincture of valerian. The author thinks that this simple plan of treatment fully deserves a further and more extensive trial.

